# Distributed gene expression modelling for exploring variability in epigenetic function

**DOI:** 10.1186/s12859-016-1313-1

**Published:** 2016-11-05

**Authors:** David M. Budden, Edmund J. Crampin

**Affiliations:** 1 Massachusetts Institute of Technology, Computer Science and Artificial Intelligence Laboratory, Cambridge, 02139 USA; 2 Systems Biology Laboratory, Melbourne School of Engineering, the University of Melbourne, Parkville, 3010 Australia; 3 ARC Centre of Excellence in Convergent Bio-Nano Science and Technology, Parkville, 3010 Australia; 4 Department of Mathematics and Statistics, the University of Melbourne, Parkville, 3010 Australia; 5 School of Medicine, the University of Melbourne, Parkville, 3010 Australia

**Keywords:** Gene expression, Epigenetics, Histone modifications, MapReduce

## Abstract

**Background:**

Predictive gene expression modelling is an important tool in computational biology due to the volume of high-throughput sequencing data generated by recent consortia. However, the scope of previous studies has been restricted to a small set of cell-lines or experimental conditions due an inability to leverage distributed processing architectures for large, sharded data-sets.

**Results:**

We present a distributed implementation of gene expression modelling using the MapReduce paradigm and prove that performance improves as a linear function of available processor cores. We then leverage the computational efficiency of this framework to explore the variability of epigenetic function across fifty histone modification data-sets from variety of cancerous and non-cancerous cell-lines.

**Conclusions:**

We demonstrate that the genome-wide relationships between histone modifications and mRNA transcription are lineage, tissue and karyotype-invariant, and that models trained on matched -omics data from non-cancerous cell-lines are able to predict cancerous expression with equivalent genome-wide fidelity.

**Electronic supplementary material:**

The online version of this article (doi:10.1186/s12859-016-1313-1) contains supplementary material, which is available to authorized users.

## Background

Computational frameworks for modelling gene expression as a function of gene-localised epigenetic features are becoming increasingly common in life sciences research. Previous studies by our lab [1–3] and others [4, 5] have leveraged the statistical power of modelling genes as observations of regulatory activity (versus variables in network-based analyses [6, 7]) to gain new insight into the function and interactions of transcription factors, histone modifications and DNA methylation. Recent applications include: inference of transcription factor roles from their respective binding motifs [8]; identification of regulatory elements responsible for differential expression patterns [9]; exploring the relationship between gene expression and chromatin organisation [2]; and comparative analysis of the transcriptome across distant species [10].

Despite the wealth of high-throughput sequencing data made available by recent large-scale consortia, previous predictive modelling studies have focused on a very small number of cell-lines (typically 1-to-3 [8, 9]) despite the obvious benefits of broader, integrative analyses. We attribute this largely to the size of sequencing data and widespread inability of published frameworks to decompose tasks into parallelisable units. Although some studies have considered accelerated GPU implementations [11], this imposes strict memory constraints and does not readily extend to large-scale, distributed systems of commodity hardware. In this study, we demonstrate how the MapReduce programming paradigm can be applied to a broad class of regression modelling that captures popular formulations of predictive gene expression modelling [1]. Importantly, we prove general asymptotic speedup in number of processing cores that is not bound to specific hardware infrastructure; i.e. cloud versus enterprise or distributed versus shared-memory multicore systems.

A recent study by Jiang et al. has suggested that RNA- (transcriptomic) and ChIP-seq (epigenetic) data generated under the same conditions (i.e. the same cell-line) introduce statistical bias and that specialised methods are necessary for accurately modeling the expression of cancer cells [12]. This study investigates both of these concerns, exploiting the computational efficiency of our distributed implementation to conduct an integrative analysis of six histone modifications across eight dissimilar ENCODE cell-lines. First, we extend our predictive modelling framework to include *L* ^2^-regularisation, which is specifically designed to prevent over-fitting to experimental noise rather than meaningful biological relationships. We then quantify the extent of condition-specific bias by training and testing models on all 64 directed, pairwise combinations of cell-lines.

## Methods

### ENCODE cell-line data

Matched mRNA transcript abundance (RNA-seq) and histone modification (ChIP-seq) data were downloaded from ENCODE [13] for the eight cell-lines summarised in Table 1. These dissimilar cell-lines are those for which data are available for the histone modifications listed in Table 2. The remaining histone modifications available from ENCODE are unsuitable for this study as they assert their functional role in non-promoter regions (e.g. H3K36me3 in the 3^′^-UTR). The MapReduce implementation of gene expression modelling presented in this study could be trivially extended to model more cell-lines if the data were made available.

**Table 1:** All ENCODE cell-lines for which matched ChIP-seq data was available for the full set of histone modifications considered in this study (listed in Table 2)

Cell-line	Tier	Description	Lineage	Tissue	Karyotype
**A549**	2	Alveolar carcinoma	Endoderm	Epithelium	Cancer
**GM12878**	1	B-lymphocyte	Mesoderm	Blood	Normal
**H1-hESC**	1	Embryonic stem cells	Inner cell mass	Embryonic stem cell	Normal
**HeLa-S3**	2	Cervical carcinoma	Ectoderm	Cervix	Cancer
**HepG2**	2	Hepatocellular carcinoma	Endoderm	Liver	Cancer
**HUVEC**	2	Umbilical vein endothelial cells	Mesoderm	Blood vessel	Normal
**K562**	1	Leukemia	Mesoderm	Blood	Cancer
**NHEK**	3	Epidermal keratinocytes	Ectoderm	Skin	Normal

**Table 2:** All histone modifications considered in this study. The remaining histone modifications available from ENCODE are unsuitable for this study as they assert their functional role in non-promoter regions (e.g. H3K36me3 in the 3^′^-UTR)

Histone modification	Regulatory role	Chromatin localisation
**H2A.Z**	Bivalency	Euchromatin
**H3K4me3**	Activator/Bivalency	Euchromatin
**H3K9ac**	Activator	Euchromatin
**H3K9me3**	Repressor	Constitutive heterochromatin
**H3K27ac**	Activator	Euchromatin
**H3K27me3**	Repressor/Bivalency	Facultative heterochromatin

### MapReduce

MapReduce is programming paradigm which adapts the map-reduce functional programming construct for distributed and fault-tolerant data processing on commodity hardware. First developed by Google [14], MapReduce is now widely adopted for parallelised processing of data on terabyte and petabyte scales. A program implemented using the MapReduce paradigm consists of a sequence, 〈*μ*_1_,*ρ*_1_,*μ*_2_,*ρ*_2_,…,*μ*_*R*_,*ρ*_*R*_〉, of mappers (*μ*_*r*_) and reducers (*ρ*_*r*_) operating over 〈key;value〉 pairs. Formally, a MapReduce program executes the steps described in Algorithm 1 on input *U*_0_ until the final reducer (*ρ*_*R*_) halts [15].



The computational benefit of MapReduce follows from its inherent parallelisability, as many instances of *μ*_*r*_ are able to process their 〈key;value〉 simultaneously (likewise with *ρ*_*r*_, although all instances of *μ*_*r*−1_ must halt before any *ρ*_*r*_ can commence). The following sections detail mapper and reducer implementations for each stage of the standard predictive gene expression modelling pipeline. For additional details on the implementation or rationale of these stages, please refer to references [1–3].

### Quantifying transcriptional regulatory interactions

The strength of association between a gene, *m*∈(1,2,…,*M*), and epigenetic feature, *n*∈(1,2,…,*N*), can be calculated from a ChIP-seq data-set specific to some cell-line/condition:

$$x_{m,n} = \sum_{\substack{r \in R_{n} \\|d(r,m)| \leq d^{*}}} \phi\left(r, m\right), $$

where *R*_*n*_ is the set of ChIP-seq reads for *n*, *d*(*r,m*) is the distance (bp) separating read *r* from the TSS of *m*, and *ϕ* maps a gene-read pair to their strength of association. The maximum bin-width, *d* ^∗^, is traditionally set to 2000 to approximate the average width of ChIP-seq binding regions. Different implementations of *ϕ* are used for histone modifications (constrained sum-of-tags) versus transcription factors (exponentially decaying affinity) due to their dissimilar ChIP-seq binding profiles [2]:

$$\phi(r,m) = \begin{cases} 1 & \text{for histone modifications}\\[0.8em] \exp{\left(-\frac{d(r,m)}{d_{0}}\right)} & \text{for transcription factors} \end{cases} $$

where hyperparameter *d*_0_ controls the strength of exponential decay for quantifying transcription factor interactions and is traditionally set to *d*_0_=5000. The resultant matrix of gene-level epigenetic scores, $\mathbf {X} \in \mathcal {R}^{M\times N}$, is then log (or arsinh)-transformed and quantile-normalised for use in a regression model.

Given ChIP-seq data for epigenetic feature *n* represented in UCSC wiggle (.WIG) format:



each column, $X_{\star, n} \in \mathcal {R}^{M} : X_{\star, n} = \text {col}_{n}\left (\mathbf {X}\right)$, of the epigenetic score matrix can be efficiently calculated using MapReduce using the procedure described in Algorithm 2. Equivalent formulations can be derived for other ChIP-seq file formats.



### Linear regression with least squares fitting

Suppose $\mathbf {X} \in \mathcal {R}^{M\times N}$ is a matrix of gene-level epigenetic scores (defined above), where *M* is the number of genes (including a unity term for model bias) and *N* is the number of epigenetic variables (*M*≫*N*). It is commonplace to model the relationship between **X** and a vector of gene expression values, $Y \in \mathcal {R}^{M}$, as follows:

$$ Y = \mathbf{X}\beta + \varepsilon, $$

where *β* parameterises the linear relationship between gene expression and local epigenetic features, and *ε* are the gene-specific errors. Such models can be fitted using ordinary least squares:

$$\begin{array}{*{20}l} \hat{\beta} &= \operatornamewithlimits{argmin}_{\beta \in \mathcal{R}^{N}} \left(|| Y - \mathbf{X}\beta ||^{2} \right)\\ &= \left(\mathbf{X}^{\top}\mathbf{X}\right)^{-1}\mathbf{X}^{\top} Y, \end{array} $$

yielding the following model-based predictions of gene expression, $\hat {Y}$:

$$ \hat{Y} = \mathbf{X}\hat{\beta}. $$

Given two general matrices, $\mathbf {A} \in \mathcal {R}^{X\times Y}$ and $\mathbf {B} \in \mathcal {R}^{Y\times Z}$, the product $\mathbf {C} \in \mathcal {R}^{X\times Z} : \mathbf {C} = \mathbf {A}\times \mathbf {B}$ can be reformulated (without loss of generality) as:

$$c_{i,k} \in \mathbf{C} : c_{i,k} = A_{i,\star}^{\top}\times B_{\star, k} $$

where:

$$\begin{array}{*{20}l} A_{i,\star} &\in \mathcal{R}^{X} : A_{i,\star} = \text{col}_{i}\left(\mathbf{A}^{\top}\right),\\ B_{\star,k} &\in \mathcal{R}^{Z} : B_{\star, k} = \text{col}_{k}\left(\mathbf{B}\right). \end{array} $$

This formulation of matrix multiplication can be implemented by the MRMultiply function defined in Algorithm 3.



Our implementation of linear regression with least squares fitting involves decomposing $\hat {\beta }$ into the product **A** ^−1^ *B*, where $\mathbf {A} \in \mathcal {R}^{N\times N} : \mathbf {A} = \text {MRMultiply}(\mathbf {X}^{\top }, \mathbf {X})$ and $B \in \mathcal {R}^{N} : B = \text {MRMultiply}(\mathbf {X}^{\top }, Y)$. The product **A** ^−1^ *B* is calculated using standard, single-processor multiplication as the communication overhead of MapReduce cannot be amortised across small matrices.

### Regularised least squares regression

Regularisation is a common method of overcoming the issue of over-fitting regression-based models to experimental noise rather than meaningful biological relationships. Regularisation involves penalising the fitted parameters, *β*, by an empirically-tuned hyperparameter, *λ*:

$$\hat{\beta} = \operatornamewithlimits{argmin}_{\beta \in \mathcal{R}^{N}} \left(|| Y - \mathbf{X}\beta ||^{2} + \lambda ||\beta||^{2} \right). $$

Presuming ||·|| is the *L* ^2^ (Euclidean) norm, our MapReduce implementation can be trivially extended to support regularisation (implementing ridge regression). Specifically, given:

$$\tilde{Y} = \left[ \begin{array}{l} Y\\ 0 \end{array} \right],\; \tilde{\mathbf{X}} = \left[ \begin{array}{l} \mathbf{X}\\ \sqrt{\lambda}I_{N} \end{array},\right] $$

where *I*_*N*_ is the *N*×*N* identity matrix, it follows that:

$$\begin{array}{*{20}l} \hat{\beta} &= \text{argmin}_{\beta \in \mathcal{R}^{N}} \left(|| \tilde{Y} - \tilde{\mathbf{X}}\beta ||^{2} \right)\\ &= \left(\tilde{\mathbf{X}}^{\top}\tilde{\mathbf{X}}\right)^{-1}\tilde{\mathbf{X}}^{\top} \tilde{Y}\\ &= \left(\mathbf{X}^{\top}\mathbf{X} + \lambda I_{n}\right)^{-1}\mathbf{X}^{\top} Y. \end{array} $$

It is evident that this implementation yields the same asymptotic time complexity as ordinary least squares regression. Moreover, the existence theorem for general ridge regression demonstrates that it is always possible to tune *λ* (e.g. using cross-validation) to reduce the mean square error of model predictions [16, 17]. This is particularly important when introducing a large number of epigenetic variables into a predictive model; e.g. a systematic analysis of the roles of dozens of transcription factors from their ChIP-seq binding profiles. In this study, *λ* is assigned the largest possible value such that the mean 10-fold cross-validated error is within 1 standard error of the minimum (solved iteratively).

Unlike the *L* ^2^ norm, the *L* ^1^ norm is often used to enforce sparsity in *β* under the assumption that most variables in **X** are physically decoupled from *Y*. This is less relevant in the context of gene expression modelling due to the well-established functional importance of epigenetic regulators for which ChIP-seq data is widely available. Moreover, the *L* ^1^ norm is not differentiable and thus not amenable to a closed-form MapReduce solution, and the parallelisation of iterative solutions is discussed elsewhere [18]. A single-node implementation of our code (see Additional file 1) is provided for convenient reproduction of our experimental results.

## Results and discussion

### MapReduce enables time-efficient gene expression modelling

For *M* genes and a ChIP-seq data-set containing *R* mapped reads, the asymptotic time complexity class of generating a column *X*_⋆,*n*_ of **X** is *Θ*(*MR*). By first preprocessing the list of gene TSS loci (invariant between epigenetic datasets) into a balanced binary search tree and observing that the vast majority of reads are within *d* ^∗^ bp of exactly zero-or-one gene, our MapReduce implementation of calculating *X*_⋆,*n*_ yields the following complexity when distributed across *P* MapReduce nodes:

$$\text{MREpigeneticScores} \in \Theta\left(\frac{R\log(M)}{P}\right), $$

which must be completed separately for each epigenetic feature, *n*∈(1,2,…,*N*).

For $\mathbf {X} \in \mathcal {R}^{M\times N}$ and $Y \in \mathcal {R}^{M}$, the asymptotic time complexity of ordinary least squares fitting $\hat {\beta } = f(\mathbf {X}, Y)$ can also be derived:



Observing that *R*≫*M*≫*N* for gene expression modelling and by distributing the calculation of **A** and *B* across *P* MapReduce nodes, the overall complexity reduces to:

$$\begin{array}{*{20}l} \text{MRExpressionModelling} &\in \Theta\left(\frac{NR\log(M)}{P}\right)\\ \end{array} $$

thus this MapReduce implementation of gene expression modelling yields an optimal *Θ*(*P*) improvement in asymptotic time complexity without the need to parallelise matrix inversion or transpose operations. The following results sections demonstrate how this improved performance can allow us to gain new insights from the large-scale integration of publicly available data-sets.

### Histone modifications are predictive of gene expression in both cancerous and normal cell-lines

**Fig. 1:**
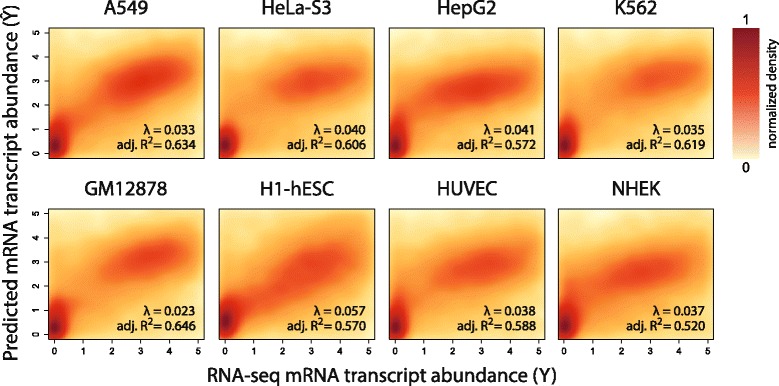
Density plots of predicted ($\hat {Y}$) versus measured (*Y*) mRNA transcript abundance abundance for cancerous (top row, mean adj. *R* ^2^=0.608) and normal cell-lines (bottom row, mean adj. *R* ^2^=0.581). The adj. *R* ^2^ performance and *λ* regularisation parameter (fitted using 10-fold cross validation) is reported for each cell-line

*L* ^2^-regularised linear regression models of genome-wide mRNA transcript abundance were constructed as functions of the following histone modifications: H2A.Z, H3K4me3, H3K9ac, H3K9me3, H3K27ac and H3K27me3. For each model, the regularisation parameter, *λ*, was fitted using 10-fold cross-validation. The adj. *R* ^2^ performance of each model is presented in Fig. 1, along with a density plot of predicted ($\hat {Y}$) versus measured (*Y*) transcript abundance. It is evident that histone modifications are accurate predictors of gene expression in both cancerous (top row, mean adj. *R* ^2^=0.608) and normal cell-lines (bottom row, mean adj. *R* ^2^=0.581), despite recent studies suggesting that specialised models are necessary to appropriately model cancerous cells [12].

**Fig. 2:**
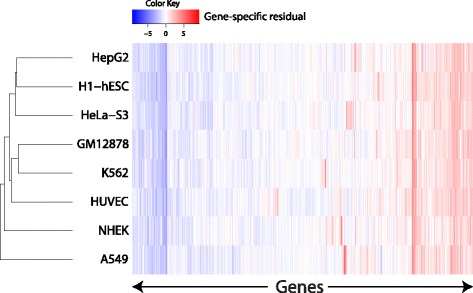
Hierarchical clustering of cell-lines by mRNA transcript abundance residuals ($\varepsilon = Y - \hat {Y}$). The three mesodermal derivatives GM12878, K562 and HUVEC cluster together, suggesting that residuals are partially non-random and instead convey meaningful biological information. Consistently, it is evident that the expression levels of many genes are poorly predicted across all eight cell-lines, presumably capturing divergence from histone modification-mediated regulation (explored in detail in our previous study [2])

Figure 2 presents the results of hierarchically clustering cell-lines by mRNA transcript abundance residuals ($\varepsilon = Y - \hat {Y}$). Interestingly, the three mesodermal derivatives GM12878, K562 and HUVEC form a distinct cluster. RNA-sequencing data for the least similar cell-line (A549) was generated at Cold Spring Harbor Laboratory whereas all other transcriptomic data was generated at the California Institute of Technology, suggesting that batch effects may be a contributing factor. It is also evident that the expression levels of many genes are consistently over- or under-estimated across all eight cell-lines. Taken together, these results indicate that gene-specific residuals are non-random and indicative of genes that are inherently difficult to model from histone modification data. The existence of genes with transcriptional activity apparently decoupled from the local epigenetic landscape has been explored in detail in our previous study [2].

### The regulatory function of histone modifications are cell-line invariant

**Fig. 3:**
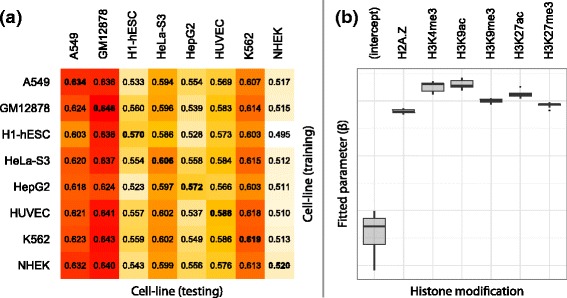
**a** Genome-wide accuracy of mRNA transcript abundance predictions (adj. *R* ^2^) for models trained and tested on each pairwise combination of cell lines. These results are strikingly non-symmetric, with significant dissimilarity between columns (*predictions*) but not rows (*training observations*). **b** Distribution of each fitted model parameter, $\hat {\beta }_{m}$, across all cell-lines considered in this study

To assess the extent to which condition-specific bias influences the reported accuracy of gene expression predictions, we trained and tested models on all 64 directed, pairwise combinations of cell-lines. The adj. *R* ^2^ performance for these models are presented in Fig. 3 a. These results demonstrate significant non-symmetry, with dissimilarity between columns (predictions) but not rows (training observations). This demonstrates that the transcriptional regulatory roles of histone modifications are cell line invariant at a genome-wide level (within the constraints of a linear model); e.g. A549 and GM12878 expression can be accurately predicted by models trained on any cell-line, despite their diversity in lineage, tissue and karyotype. These results are further supported by Fig. 3 b, which demonstrates consistency in the fitted model parameters, $\hat {\beta }$, across all cell-lines.

It is worth noting that that models trained and tested using data from a single cell-line (boldfaced along the diagonal of Fig. 3 a) only marginally outperform models trained on dissimilar cell-lines and, moreover, that these margins are significantly less than the inherent variation between columns. These findings suggest that, in the context of gene expression modelling, training and testing models on data generated under the same experimental conditions (i.e. the same cell-line) is not a significant source of statistical bias.

## Conclusions

Many previous predictive modelling studies have been limited in scope to 1-3 cell-lines due to the computational expense of modelling high-throughput sequencing data. In this study, we introduced a MapReduce implementation of gene expression modelling that is able to obtain a full *Θ*(*P*) improvement in asymptotic time complexity when distributed across *P* CPUs (e.g. as part of multi-core PC or high-performance cluster). This formulation and corresponding complexity analysis is intended to demonstrate the minimal set of operations that should be parallelised to yield *Θ*(*P*) improvement. Practically, machine learning pipelines implemented in TensorFlow [19], FlumeJava [20] or similar technologies would minimise execution time on conventional hardware without the added difficulty of implementing mappers and reducers. For illustrative purposes, a pure MapReduce implementation was applied in this study to model more than 50 epigenetic and matched transcriptomic data-sets across 8 dissimilar ENCODE cell-lines. We encourage other researchers to investigate similar optimisations to increase the volume of data modelled in future integrative analyses.

Despite recent studies presenting specialised methods for modelling cancerous gene expression [12], we find no evidence of variation in the statistical relationship between histone modifications and mRNA transcript abundance in normal-versus-cancerous cell-lines. Although our results demonstrate that some cell-lines are inherently more difficult to model than others, this trait appears to be more closely associated with the extent of cellular differentiation than carcinogenic state; e.g. models of h1-hESC embryonic stem cells perform 12 % worse than terminally-differentiated GM12878 lymphoblasts. Although the NHEK (Normal Human Epidermal Keratinocytes) cell-line is both terminally-differentiated and exhibits the worst-performing models, this may be attributed to the phenotypic plasticity of keratinocytes between epithelial and mesenchymal states (necessary for wound healing). We therefore speculate that the predictability of a cell-line’s genome-wide expression levels from epigenetic data is proportional to its transcriptomic rigidity; i.e. cells with signal-induced phenotypic plasticity are less likely to exhibit a stable, predictive epigenome.

Interestingly, hierarchical clustering of the 8 investigated cell-lines by mRNA transcript abundance residuals (gene-level prediction errors) was able to group the closely-related, mesodermal-derivative cell-lines GM12878, K562 and HUVEC; again, carcinogenic state appeared to have little effect on the propensity of two cell-lines to cluster together. Taken together with the observation that many genes exhibited large and consistent residuals across all cell-lines, these results suggest that gene-level residuals are non-random and, moreover, that the transcriptional activity of many genes are decoupled from their local epigenetic landscape. These observations are consistent with and extend upon the findings of our earlier studies [2, 3], and we hope that future studies will leverage distributed computational modelling to further accelerate progress in this field.

## Additional file

Additional file 1 A single-node implementation of our code is provided for convenient reproduction of our experimental results. (ZIP 363 kb)

## Abbreviations

ChIP: Chromatin immunoprecipitation
ChIP-seq: ChIP with massively parallel sequencing
GRN: Gene regulatory network
H1/2A/2B/3/4: Histone proteins
H2A.Z: Histone H2 variant
hESC: Human ESC
H*x*K*yz*: Modification *z* of lysine *y* on histone H*x*
mRNA: Messenger RNA
RNA: Ribonucleic acid
RNA-seq: High-throughput RNA sequencing
TSS: Transcription start site
