# Supplementary figures and images for: Distributed gene expression modelling for exploring variability in epigenetic function
Source: BMC Bioinformatics. 2016 Nov 5;17:446. doi: 10.1186/s12859-016-1313-1 (PMC5097851; doi:10.1186/s12859-016-1313-1)

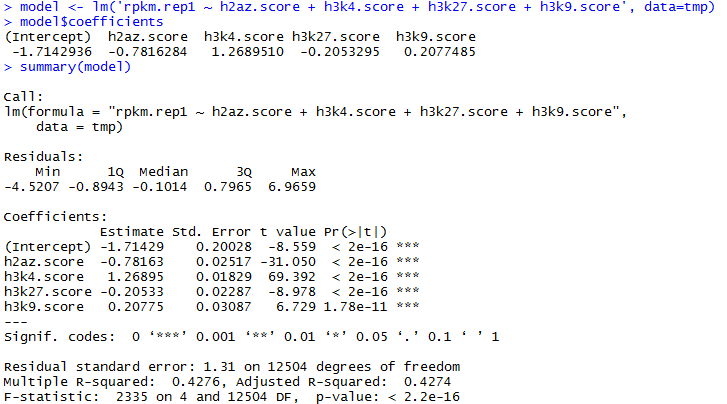

Supplement: Additional file 1 — A single-node implementation of our code is provided for convenient reproduction of our experimental results. (ZIP 363 kb) [file 12859_2016_1313_MOESM1_ESM.zip › Code/expected_output.png]
